# Density Functional Theory Study of Low-Dimensional (2D, 1D, 0D) Boron Nitride Nanomaterials Catalyzing Acetylene Acetate Reaction

**DOI:** 10.3390/ijms23179997

**Published:** 2022-09-02

**Authors:** Xunchao Zhang, Lihua Kang, Mingyuan Zhu

**Affiliations:** 1 College of Chemistry & Chemical Engineering, Yantai University, Yantai 264005, China; 2 School of Chemistry & Chemical Engineering, Shihezi University, Shihezi 832000, China

**Keywords:** acetylene, acetic acid, vinyl acetate, BN nanomaterial, dimension

## Abstract

In this paper, density functional theory (DFT) was used to study the possibility of low-dimensional (2D, 1D, 0D) boron nitride nanomaterials to catalyze acetylene acetate reaction, and further explore the possible source of this catalytic activity. It is found that the catalytic activity of boron nitride nanomaterials for acetylene acetate reaction will change with the change of the geometric structure (dimension) and reaction site of the catalyst. From the geometric structure, the reaction components and the zero-dimensional BN catalyst can form chemical bonds and form complexes, while only physical adsorption occurs on the surface of the one-dimensional and two-dimensional BN catalysts. From the reaction site, the properties of different C sites on the B_12_N_12_NC-C_2_H_2_ complexes are different. Namely, a C atom connected with a B atom is more likely to have an electrophilic reaction with H^+^, and a C atom connected with an N atom is more likely to have a nucleophilic reaction with CH_3_COO^−^. Through the study of three kinds of BN nanomaterials with low dimensions, we found that the zero-dimensional B_12_N_12_ nanocage broke the inherent reaction inertia of BN materials and showed good catalytic activity in an acetylene acetate reaction, which is very likely to be a non-metallic catalyst for the acetylene gas-phase preparation of vinyl acetate.

## 1. Introduction

As one of the most-used industrial organic raw materials in the world, vinyl acetate (VAc) can produce derivatives such as polyvinyl acetate resin (PVAc), polyvinyl alcohol (PVA), and polyacrylonitrile (PAN) through self-polymerization or copolymerization with other monomers. These derivatives are widely used in construction, textile, machinery, medicine, and soil amendment [1,2,3]. Due to the rapid development of the terminal industry, the demand for vinyl acetate is increasing year by year [4]. The research and development of new catalysts will become one of the key factors to improve the production capacity and yield of vinyl acetate. At present, due to the influence of raw materials and processes, the production of vinyl acetate is mainly based on the ethylene gas phase method and acetylene gas phase method. Compared with the acetylene method, the ethylene gas phase method has more advanced technology and higher production efficiency. However, in China, India, and other countries with rich coal resources and scarce oil resources, the acetylene process is more competitive than the ethylene process in the raw material side [5]. Although the production capacity of the vinyl acetate industry has been greatly increased in recent years, the technology used in the acetylene process in industry is still relatively basic. In addition, the traditional zinc acetate catalyst used in the acetylene method also has some disadvantages, such as rapid decline in activity, more by-products, easy carbon deposition in the reaction process, and easy sintering of active components [6,7,8]. Therefore, the research and development of new catalysts is conducive to improving the current situation of the acetylene process, and has important practical significance for the healthy development of the vinyl acetate industry and the improvement of production capacity in areas rich in coal resources.

In light of today’s increasingly serious environmental problems, developing efficient, cheap, and environmentally friendly green catalysts is an effective means to achieve the goal of “carbon neutrality” and sustainable development strategy. Because the properties of the elements close to each other in the periodic table are similar, it inspires us to look for new substances to replace the traditional zinc-based catalysts in a certain area of the periodic table. The outer electron arrangement of the Zn element in zinc-based catalysts is 3d^10^4s^2^. Because the 10-electron structure of the 3d orbital of Zn is very stable, it is very difficult to lose the electrons in the 3d orbital in chemical reactions [9]. This shows that Zn-based catalysts rely on 4s orbitals in the process of catalytic acetylation, while the “d-band theory” [10] based on transition metals is not applicable to Zn-based catalysts. Therefore, we speculate that the boron group (IIIA group), which is adjacent to the zinc group (IIB group) elements, may be the best substitute for zinc-based catalysts. Among the boron group (IIIA group) elements, boron, as the only non-metallic material, has attracted the extensive attention of researchers because of its low cost and environmental friendliness. Boron is electron deficient and can carry empty orbitals and occupy orbitals. At the same time, similar to transition metal elements, boron has the ability to attract electrons (pull) and give electrons (push) at the same time, so it shows unique activity in many catalytic reactions [11]. As an important compound form of boron, boron nitride exists in a variety of structures, such as three-dimensional cubic and hexagonal phase bulk boron nitride [12], two-dimensional h-BN nanosheets [13,14], one-dimensional BN nanotubes [14], and zero-dimensional BN nanocages [15]. Boron nitride compounds usually have good stability of oxidation resistance, heat resistance, and corrosion resistance, and have great advantages in industrial applications. In particular, BN nanomaterials have shown broad application prospects in energy storage, catalysis, molecular sensing, tribology, heat transport, and drug transport [16,17,18,19,20]. Therefore, BN nanomaterials are very desirable for catalytic reactions. These excellent properties also make it possible to prepare catalysts with strong toxicity resistance, good thermal stability, and long service life. Because B and N atoms in BN compounds usually form sp^2^ or sp^3^ hybridization, the p orbital of the B atom is completely filled, which is usually considered to be chemically inert. In addition to introducing defects [21] and edges [22], heteroatom doping [23], and chemical functionalization [24,25] to activate BN nanostructures, studies have shown that reducing the dimension of materials can produce strange electronic structures, and can obtain a larger surface area and higher atom utilization efficiency [11], which may be one of the potential ways for inert BN materials to obtain catalytic properties.

**Figure 1:**
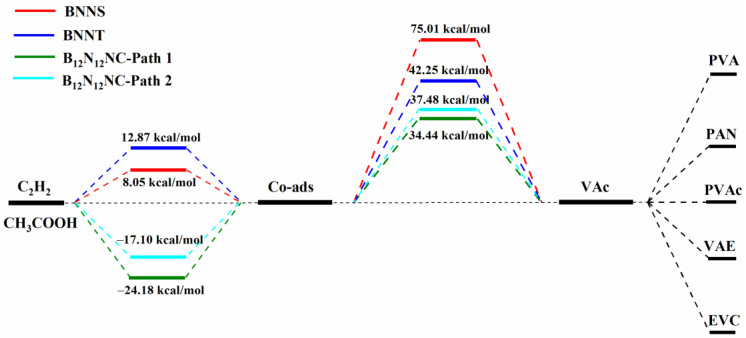
Energy change of acetylene acetic acid reaction catalyzed by BN catalysts with different geometric structures at 1 atm and 493.15 K.

In order to explore whether the catalytic activity of boron nitride nanomaterials for acetylene acetate reaction will change with the geometric structure and dimension of the catalyst, we used density functional theory (DFT) to study the possibility of low-dimensional BN nanomaterials (BN nanosheet (2D), boron nanotube (1D), and boron nanocage (0D)) for acetylene acetate reaction, and further discussed the differences of the three catalysts and the source of their activity. Figure 1 briefly describes the change of energy of acetylene acetylation catalyzed by three BN catalysts. The results show that under the reaction conditions of 1 atm and 493.15 K, the adsorption of acetylene and acetic acid molecules on BN nanosheets and BN nanotubes is thermodynamically unfavorable. However, with the decrease of the dimension of BN nanomaterials, the adsorption capacity of the 0D B_12_N_12_ nanocage for acetylene and acetic acid was significantly enhanced, and it showed good catalytic activity in the reaction of acetylene and acetic acid. This shows that the electronic properties of the surface of BN nanomaterials can be effectively adjusted and controlled by reducing the dimension of BN nanomaterials, so that inert materials can produce catalytic activity. The research of this paper shows that the 0D B_12_N_12_ nanocage is likely to be a potential non-metallic catalyst for the preparation of vinyl acetate by the acetylene gas phase method. We hope this work can provide theoretical support for the research and development of non-metallic catalysts for acetylene acetate reaction, and provide theoretical help for the research of new low-dimensional nano catalysts.

## 2. Results and Discussion

### 2.1. Analysis of Structure and Properties of Catalysts

**Figure 2:**
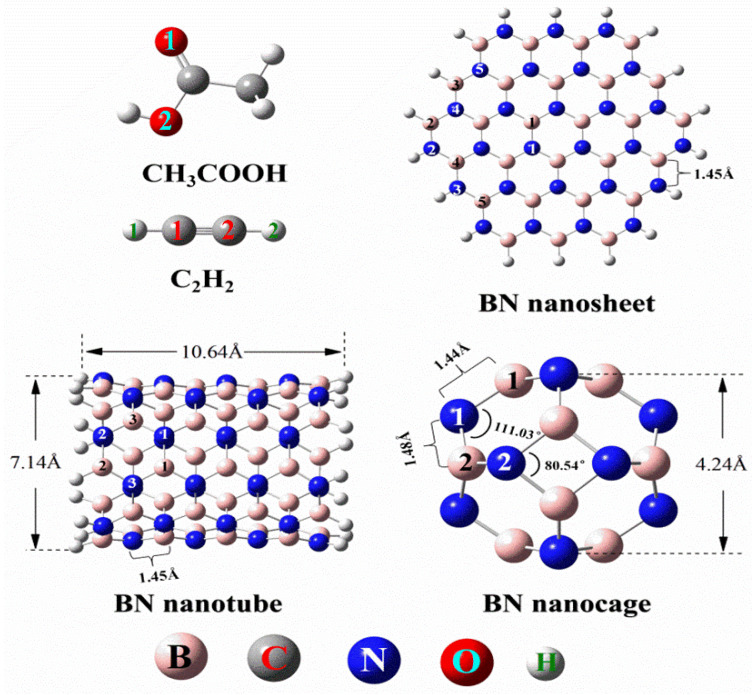
Optimized structures of C_2_H_2_, CH_3_COOH and BN nanosheet, BN nanotube, BN nanocage. (Pink is B atom, gray is C atom, blue is N atom, red is O atom, white is H atom).

The molecular geometric structures of BN nanosheets, BN nanotubes, B_12_N_12_ nanocages, acetylene, and acetic acid after optimization are shown in Figure 2. Hexagonal boron nitride nanosheets (BNNS) have the same hexagonal structure as graphene. Due to their similar crystal structure, single-layer BN nanosheets have many properties similar to graphene, but are white in color, so they are sometimes called white graphene [16]. In particular, the hexagonal boron nitride monolayer, in which boron and nitrogen atoms alternate in a honeycomb vortex, consists of two-dimensional (2D) layers bound by sp^2^ bonds [26]. In each layer of the hexagonal boron nitride, boron and nitrogen atoms are held together by strong covalent bonds, and the layers are held together by weak van der Waals forces, just as in graphite [25]. We constructed and optimized the single-layer nanosheet structure of BNNS through theoretical simulation, where each alternate B-N bond length is 1.45 Å and each Angle ∠B-N-B or ∠N-B-N is 120°. The Mulliken charges of the B atom and N atom are 0.482(B^1^), −0.477(N^1^), 0.334(B^2^), −0.521(N^2^), 0.349(B^3^), −0.536(N^3^), 0.478(B^4^), −0.431(N^4^), 0.478(B^5^), and −0.432(N^5^), respectively. The corresponding atomic serial numbers are shown in Figure 2.

Boron nitride nanotubes (BNNT) are a seamless cylindrical hexagonal BN bond grid composed of alternate boron and nitrogen atoms. Although BNNTs and carbon nanotubes (CNT) have similar structures, BNNTs also exhibit a series of physical and chemical properties that are different from those of CNT, mainly due to the partial ionic bond properties between the B and N atoms [27]. In addition, the boron–nitrogen bonds of BNNT have certain polar characteristics, depending on the curvature of the nanotubes. With the increase of tube curvature, the sp^2^ hybridization of the B atom and N atom in the large-diameter BNNS changes to the sp^3^ hybridization in the small-diameter BNNT. This has important implications for the nature of interactions between functional molecules and BNNT walls [28]. We constructed and optimized the monolayer structure of the BN nanotube by theoretical simulation. The model consists of 40 N atoms, 40 B atoms, and 20 H atoms. The length and diameter of the nanotube are 10.64 Å and 7.14 Å. Each of the alternate B-N bonds is 1.45 Å long, and each of the ∠B-N-B or ∠N-B-N is 120°. The Mulliken charges of the B atom and N atom are 0.465(B^1^), −0.465(N^1^), 0.332(B^2^), −0.525(N^2^), 0.493(B^3^), and −0.44(N^3^), respectively (the corresponding atomic serial numbers are shown in Figure 1). The Mulliken charges of the B atom and N atom are 0.465(B^1^), −0.465(N^1^), 0.332(B^2^), −0.525(N^2^), 0.493(B^3^), and −0.44(N^3^), respectively.

Oku et al. [29,30,31] have successfully synthesized a series of cage-like BN nanoclusters of different sizes and shown that of these clusters, fullerene-like B_12_N_12_ cages are the most energy-stable BN nanoclusters. Since then, the geometry and energy of the B_12_N_12_ nanocages have been extensively studied. For B_12_N_12_ nanocages (B_12_N_12_NC), the ground state geometry represents a hollow cage with six quadrilateral and eight hexagons, arranged in tetrahedron (Th symmetry) [32]. It consists of 36 covalent bonds between B and N atoms formed by sp^2^ hybridization. The distance between the two quadrilaterals in BN nanocages is 4.24 Å, and there are two different B-N bonds in B_12_N_12_NC. The length of the B^1^-N^1^ bond is 1.44 Å, and that of the B^2^-N^1^ bond is 1.48 Å. In the hexagonal BN ring, ∠B^1^ with atom B as its vertex is 110.03°, and in the quadrilateral ring with atom B^2^ as its vertex, ∠B^2^ is 80.54°. These bond lengths and bond angles are very consistent with previous studies [33,34]. The Mulliken charges of the B atom and N atom in the catalyst were 0.440(B) and −0.440(N), respectively. The Mulliken charges of the B atom and N atom in the catalyst were 0.440(B) and −0.440(N), respectively.

**Figure 3:**
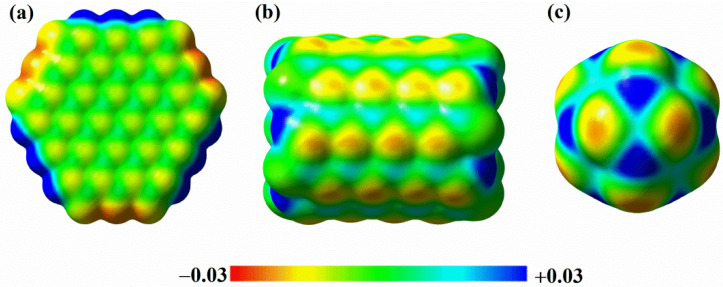
Molecular surface electrostatic potential of BN nanosheets (**a**), BN nanotubes (**b**), and BN nanocages (**c**) (electron density: 0.001 electrons/bohr^3^).

Without considering the atoms at the boundary, we can find that the Mulliken charges of the B and N atoms on the three BN nanomaterials are similar. However, according to the molecular surface electrostatic potential shown in Figure 3 (the high electron density is close to the red region and the low electron density is close to the blue region), it can be seen that the electrostatic potential on the molecular surface of different BN nanomaterials is obviously different, and the difference of electron cloud density on the surface of B_12_N_12_ nanocages is the most obvious. This shows that the change of the BN nanomaterial structure will affect the electron cloud density on the molecular surface. The good charge transfer on the surface of B_12_N_12_ nanocages also enables us to see the potential of breaking the chemical inertia on the surface of the BN material, providing the possibility of developing highly active BN catalysts.

### 2.2. Adsorption of C_2_H_2_ and CH_3_COOH on BN Nanomaterials with Different Structures

**Figure 4:**
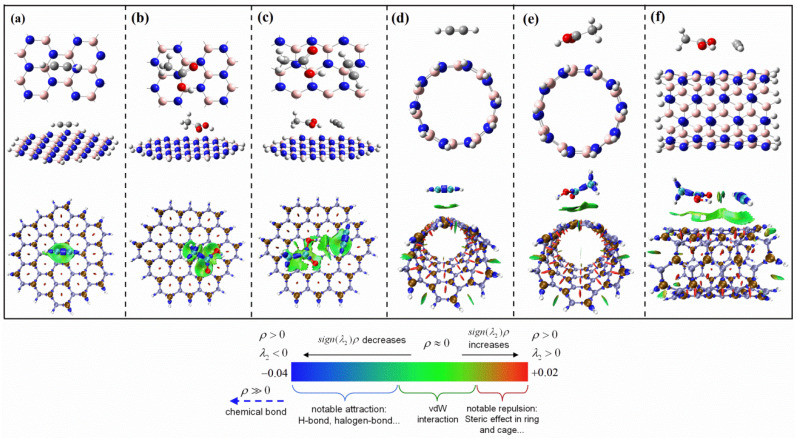
Optimized adsorption structures of C_2_H_2_ and CH_3_COOH molecules on BN nanomaterials and the corresponding interaction region indicator (IRI) analysis ((**a**–**c**) are the single adsorption and co-adsorption structures of C_2_H_2_ and CH_3_COOH molecules on BNNS, respectively; (**d**–**f**) are the single adsorption and co-adsorption structures of C_2_H_2_ and CH_3_COOH molecules on BNNT, respectively).

First, we analyzed the adsorption of CH_3_COOH and C_2_H_2_ on BNNS. The most stable single adsorption and co-adsorption configurations are shown in Figure 4a–c, respectively. Under the reaction conditions of 493.15 K and 1 atm, the adsorption energy of C_2_H_2_ on BNNS is 2.68 kcal/mol. The adsorption energy of CH_3_COOH is 4.57 kcal/mol, and the co-adsorption energy of CH_3_COOH and C_2_H_2_ molecules on BNNS is 8.05 kcal/mol. Figure 4d–f show the single adsorption and co-adsorption structures of C_2_H_2_ and CH_3_COOH on BNNT, respectively. Under the reaction conditions of 493.15 K and 1 atm, the adsorption energy of C_2_H_2_ on BNNT is 4.39 kcal/mol, the adsorption energy of CH_3_COOH on BNNT is 7.65 kcal/mol, and the co-adsorption energy of CH_3_COOH and C_2_H_2_ molecules on BNNT is 12.87 kcal/mol. In addition, we also studied the adsorption performance of the reaction components at the boundary of the hydrogen atom-enclosed BNNS and BNNT. The specific adsorption energy and the optimized adsorption configuration are shown in Table S1 and Figure S1 in the Supporting Information. Since the boundary position has little influence on the adsorption energy of the reaction components, the subsequent research is carried out on the catalyst surface. During the adsorption process on the catalyst surface, the structure of reactant molecules adsorbed on BNNS and BNNT has almost no obvious change, which indicates that the influence of the catalyst on reactants may be relatively weak. As shown in Figure 4, we conducted corresponding interaction region indicator (IRI) analysis [35] on the adsorption structure. If the IRI isosurface is green, it indicates that this is the van der Waals action region; if the color of the isosurface is red, it indicates that there is a certain steric effect here; if the isosurface color is blue, it indicates that there is a significant attraction. It can be seen from Figure 4 that the interaction force between the reactant and catalyst is mainly van der Waals force, which indicates that the adsorption of acetylene and acetic acid molecules on BNNS or BNNT is essentially physical adsorption. As physical adsorption depends on temperature, taking the simplest Langmuir adsorption model as an example, the adsorption amount of physical adsorption will decrease significantly with the increase of temperature. When the reaction conditions are 493.15 K, 1 atm, the adsorption energy of reactants on BNNS and BNNT is positive, which also shows that the adsorption of reactants by BNNS and BNNT is thermodynamically unfavorable under industrial reaction conditions. 

**Figure 5:**
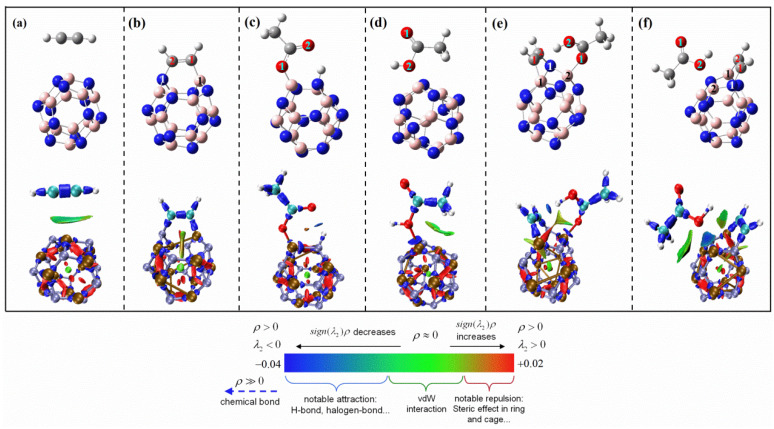
Optimized adsorption structures of C_2_H_2_ and CH_3_COOH molecules on B_12_N_12_ nanocages and the corresponding interaction region indicator (IRI) analysis ((**a**,**b**) are the single adsorption structures of C_2_H_2_ on BNNC, respectively; (**c**,**d**) are the single adsorption structures of CH_3_COOH on BNNC, respectively; (**e**,**f**) are the co-adsorption structures of C_2_H_2_ and CH_3_COOH on BNNC, respectively).

With the change of structure, the adsorption mode of the B_12_N_12_ nanocages on reactants also changed significantly. Under the reaction conditions of 493.15 K and 1 atm, different adsorption configurations and IRI analysis between reactants and adsorption structures are shown in Figure 5. In Figure 5a, the adsorption energy of C_2_H_2_ on B_12_N_12_NC is 5.32 kcal/mol, but in Figure 5b, the adsorption energy of C_2_H_2_ on B_12_N_12_NC is −26.33 kcal/mol. According to IRI analysis, the interaction between acetylene and B_12_N_12_NC in Figure 5a depends on the van der Waals force, which is a typical physical adsorption; the chemical bond between acetylene and B_12_N_12_NC in Figure 5b belongs to chemical adsorption. By comparing the adsorption energy and bonding mode, it can be concluded that the most favorable adsorption mode of acetylene on B_12_N_12_NC is the structure in Figure 5b. Compared with the physical adsorption on BNNS and BNNT, the B-N bond at the adsorption site breaks during the chemical adsorption of acetylene molecules on B_12_N_12_NC, and a B_12_N_12_NC-C_2_H_2_ complex is formed between acetylene and B_12_N_12_NC. In this process, the C≡C triple bond (sp hybrid) of acetylene is transformed into a C=C double bond (sp^2^ hybrid), which makes acetylene molecules activated and polarized, thus improving the reaction activity with acetic acid molecules. In Figure 5c, the adsorption energy of CH_3_COOH on B_12_N_12_NC is −19.43 kcal/mol, and in Figure 5d, the adsorption energy of CH_3_COOH on B_12_N_12_NC is 6.27 kcal/mol. IRI analysis showed that the difference of two adsorption structures of acetic acid on B_12_N_12_NC was due to the interaction between two different types of O on carboxyl group and B on catalyst. In Figure 5c, acetic acid is dissociated and adsorbed during the adsorption process. The O^1^ atom on the carboxyl group forms a B-O bond with the B atom, and the H atom on the carboxyl group forms an N-H bond with the N atom. In Figure 5d, the acetic acid is not dissociated during the adsorption process, but the O^2^ atom on the carboxyl group interacts with the B atom on the catalyst. It can be seen from IRI analysis that chemical bonds have also been formed between O^2^ and B at this time, but compared with the adsorption energy of acetic acid dissociation adsorption in Figure 5c, the adsorption in Figure 5d is thermodynamically unfavorable. Since the adsorption energy of acetylene is lower than that of acetic acid, acetylene will be preferentially adsorbed on the catalyst. The co-adsorption energy of acetylene and acetic acid obtained on this basis is shown in Figure 5e,f. The co-adsorption energy of the two structures is −24.28 kcal/mol and −17.10 kcal/mol, respectively. The difference in adsorption energy is mainly related to the interaction between different O atoms and B atoms in CH_3_COOH. By comparing the adsorption energy of the two structures, it can be found that the adsorption energy of the structure (e) in Figure 5 is lower and more stable.

### 2.3. Reaction Mechanism of Acetylene Acetotization Catalyzed by BN Nanomaterials with Different Structures

#### 2.3.1. Reaction Process of Acetylene Acetylation on BN Nanosheets

**Figure 6:**
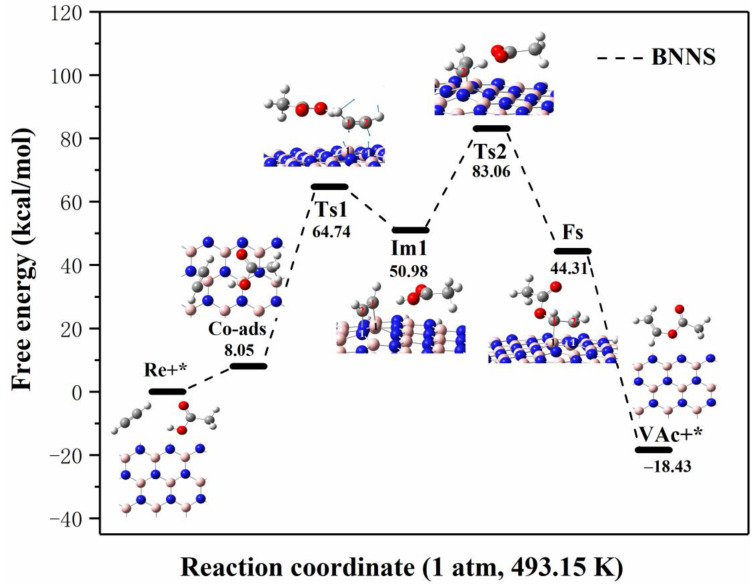
Reaction process and energy change of C_2_H_2_ and CH_3_COOH on BN nanosheets at 1 atm and 493.15 K (Re stands for reactant, * stands for catalyst, Co-ads stands for co-adsorption structure, Ts stands for transition state, Im stands for intermediate state, Fs stands for final state structure, VAc stands for vinyl acetate).

The reaction path and specific energy change process of acetylene and acetic acid molecules on boron nitride nanosheets (BNNS) are shown in Figure 6. Since the adsorption energy of acetylene and acetic acid on BNNS is positive, in order to ensure the occurrence of the reaction, the reactants must be stably fixed on the catalyst surface. Based on the co-adsorption structure, we obtained the transition state (Ts1) structure through transition state search to further generate a more stable configuration (Im1). In the transition structure (Ts1), acetylene molecule changes into vinyl cation, C^1^-C^2^ bond length changes from 1.206 Å to 1.255 Å, C^1^-H^1^ bond length extends from 1.066 Å to 1.076 Å, and C^2^-H^2^ bond length extends from 1.066 Å to 1.070 Å. Through vibrational analysis, it is found that Ts1 has a unique virtual frequency (−616.90 cm^−1^), and the vibration direction of the virtual frequency is the relative motion between C atoms on vinyl and B and N atoms on catalyst. The specific vibration direction is shown in Ts1 in Figure 6. Subsequently, the B^1^-N^1^ bond on the catalyst is broken (the bond length is lengthened from 1.445 Å to 1.710 Å), the C atom on the vinyl cation is bonded with the unsaturated B^1^ atom and N^1^ atom, respectively (C^1^-B^1^ bond length is 1.636 Å, C^2^-N^1^ bond length is 1.519 Å), and finally, the intermediate (Im1) structure is formed. During the process from Co-ads to Ts1, the system needs to absorb 56.69 kcal/mol of energy, and then the system releases 13.76 kcal/mol of energy from Ts1 to Im1. However, there is a high reaction energy barrier of 56.69 kcal/mol just at the step of forming Im1, which is very difficult to exceed, and it also makes it difficult for subsequent reactions to occur.

In order to explore the complete reaction process, we further searched for the second transition state (Ts2) based on the intermediate (Im1). The O-H bond in CH_3_COOH in Ts2 was broken (from 0.977 Å to 1.217 Å) to form H^+^ and CH_3_COO^−^, and the C^2^-N^1^ bond in BNNS-C_2_H_2_ complexes was weakened (from 1.519 Å to 1.569 Å) to form BNNS-C_2_H_2_^+^ complexes. In the process of forming vinyl acetate, H^+^ dissociated from acetic acid attacks C^2^ atom attached to BNNS-C_2_H_2_^+^ to form BNNS-C_2_H_3_^+^ complexes, and the O^1^ atom on CH_3_COO^−^ attacks the C^1^ atom of BNNS-C_2_H_3_^+^ complexes to form the final state (Fs) structure. During the process from Im1 to Ts2, the system needs to absorb 32.08 kcal/mol of energy, and then the system releases 38.75 kcal/mol of energy from Ts2 to Fs. Similarly, vibrational analysis shows that Ts2 has only one virtual frequency (−1496.39 cm^−1^), which is related to the stretching vibration of acetic acid-dissociated H^+^ between the C^1^ and O^2^ atoms. Finally, the product (VAc) is desorbed from BNNS, and the desorption energy is −62.74 kcal/mol. After VAc desorption, the structure of the BNNS recovered and continued to catalyze a new round of reactions. By comparing the reaction path and energy change in Figure 6, we can find that although the acetylene acetate reaction is exothermic in terms of total energy, the system needs to absorb 83.06 kcal/mol of energy in the process from the initial structure (Re+*) to the highest energy point (Ts2). Such a huge energy is difficult to meet under the reaction condition of 493.15 K, which further breaks the possibility of reaction. Based on the method of determining the rate-control step proposed by Murdoch et al. [36], the process from the initial structure (Re+*) to Ts2 is the decisive step of the acetylene acetate reaction catalyzed by BNNT, and the free-energy barrier of the rate-control step is 83.06 kcal/mol.

#### 2.3.2. Reaction Process of Acetylene Acetylation on BN Nanotubes

**Figure 7:**
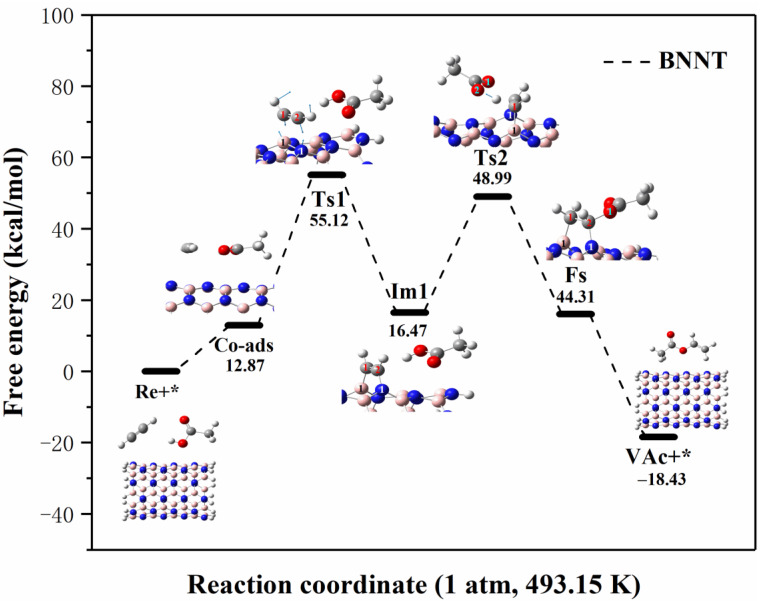
Reaction process and energy change of C_2_H_2_ and CH_3_COOH on BN nanotubes at 1 atm and 493.15 K (Re stands for reactant, * stands for catalyst, Co-ads stands for co-adsorption structure, Ts stands for transition state, Im stands for intermediate state, Fs stands for final state structure, VAc stands for vinyl acetate).

The reaction path and specific energy change process of acetylene and acetic acid molecules on boron nitride nanotubes (BNNT) are shown in Figure 7. Since the adsorption energy of acetylene and acetic acid on BNNT is similar to that on BNNS, it is also necessary to stabilize the reactants on the catalyst surface in order to ensure the reaction. Firstly, the transition state (Ts1) structure was found based on the co-adsorption structure to further generate a more stable intermediate (Im1) configuration. In the transition structure (Ts1), acetylene molecule changes into vinyl cation, the C^1^-C^2^ bond length changes from 1.206 Å to 1.259 Å, the C^1^-H^1^ bond length extends from 1.066 Å to 1.077 Å, and the C^2^-H^2^ bond length changes from 1.066 Å to 1.073 Å. Through vibrational analysis, it is found that Ts1 has a unique virtual frequency (−549.86 cm^−1^). The vibration direction of the virtual frequency is the relative motion between the C atoms on vinyl and the B and N atoms on BNNT. The specific vibration direction is shown in Ts1 in Figure 6. During the process from Ts1 to the intermediate (Im1) structure, the B^1^-N^1^ bond on the catalyst is broken (the bond length is lengthened from 1.442 Å to 2.566 Å), and the C atom on the vinyl cation forms chemical bonds with unsaturated B^1^ and N^1^ atoms, respectively (the C^1^-B^1^ bond length is 1.574 Å and the C^2^-N^1^ bond length is 1.437 Å). During the process from Co-ads to Ts1, the system needs to absorb 42.25 kcal/mol of energy, and then the system releases 38.65 kcal/mol of energy from Ts1 to Im1. In the same reaction process, the reaction energy barrier of the first step on BNNT is 14.44 kcal/mol lower than that of the first step on BNNS, which further indicates that the change of geometric structure can change the catalytic activity of BN nanomaterials.

In order to explore the complete reaction process, we further searched for the second transition state (Ts2) based on the intermediate (Im1). In Ts2, the O-H bond in CH_3_COOH was broken (from 0.977 Å to 1.305 Å) to form H^+^ and CH_3_COO^−^, and the C^2^-N^1^ bond in BNNT-C_2_H_2_ complexes was slightly prolonged (from 1.437 Å to 1.466 Å). In the process of forming vinyl acetate, H^+^ dissociated from acetic acid attacks C^2^ atom attached to BNNT-C_2_H_2_ to form BNNT-C_2_H_3_^+^ complexes, and the O^1^ atom on CH_3_COO^−^ attacks the C^1^ atom of BNNT-C_2_H_3_^+^ complexes to form the final state (Fs) structure. During the process from Im1 to Ts2, the system needs to absorb 32.52 kcal/mol of energy, and then the system releases 4.68 kcal/mol of energy from Ts2 to Fs. Similarly, vibrational analysis shows that Ts2 has only one virtual frequency (−1379.74 cm^−1^), which is related to the stretching vibration of acetic acid-dissociated H^+^ between the C^1^ and O^2^ atoms. Finally, the product (VAc) was desorbed from BNNT, and the desorption energy was −62.74 kcal/mol. By comparing the reaction path and energy changes in Figure 6, it is found that the system needs to absorb 55.12 kcal/mol of energy from the outside to make the reaction take place in the process of acetylene acetate acidification from the initial structure (Re+*) to the highest energy point (Ts1). Based on the method of determining the rate-control step proposed by Murdoch et al., the process from the initial structure (Re+*) to Ts1 is the decisive step of the acetylene acetate reaction catalyzed by BNNT, and the free-energy barrier of the rate-control step is 55.12 kcal/mol.

#### 2.3.3. Reaction Process of Acetylene Acetylation on B_12_N_12_ Nanocages

**Figure 8:**
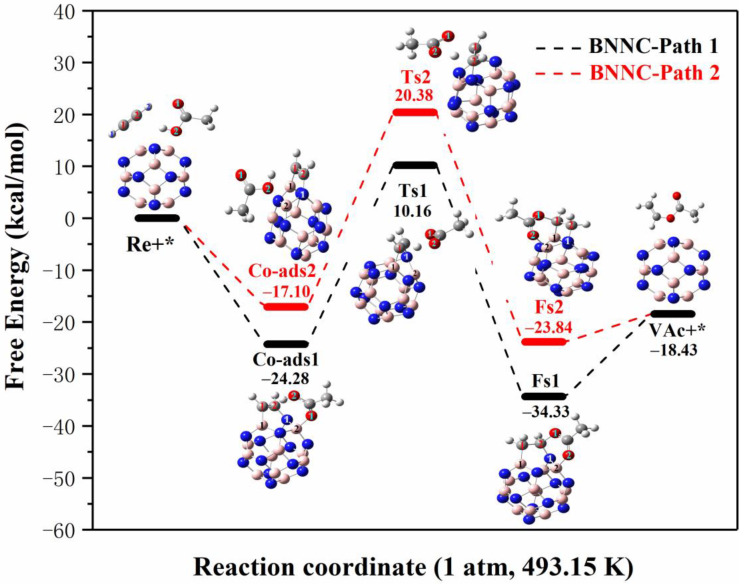
Reaction process and energy change of C_2_H_2_ and CH_3_COOH on BN nanotubes at 1 atm and 493.15 K (the black line is path 1 and the red line is path 2; Re stands for reactant, * stands for catalyst, Co-ads stands for co-adsorption structure, Ts stands for transition state, Im stands for intermediate state, Fs stands for final state structure, VAc stands for vinyl acetate).

The reaction path and specific energy change process of acetylene and acetic acid molecules on B_12_N_12_NC are shown in Figure 8. Since the adsorption of acetylene on B_12_N_12_NC is a spontaneous chemical adsorption, it makes the reaction process of acetylene acetate acidification on B_12_N_12_NC simpler and easier to occur. Since there are two co-adsorption structures on B_12_N_12_NC, we studied the specific reaction paths of the two structures, respectively. In path 1, we found the transition state (Ts1) structure connecting Co-ads1 and Fs1 through transition state search. During the formation of Ts1 from Co-ads1, the C^1^-C^2^ bond length of C_2_H_2_ changes from 1.350 Å to 1.429 Å, the O-H bond on the carboxyl group of acetic acid lengthens (from 1.002 Å to 1.254 Å) to form free H^+^ between O^2^ and C^1^, and the B^2^-O^1^ bond between acetic acid and catalyst breaks to form free CH_3_COO^−^. The H^+^ dissociated from acetic acid attacks the C^1^ atom on BNNT-C_2_H_2_ to form BNNT-C_2_H_3_^+^ complexes, and the O^1^ atom on CH_3_COO^−^ attacks the C^2^ atom on BNNT-C_2_H_3_^+^ to form the final state (Fs) structure. During the process from Co-ads1 to Ts1, the system needs to absorb 34.44 kcal/mol of energy, and then the system releases 44.49 kcal/mol of energy during the process from Ts1 to Fs1. Vibrational analysis shows that Ts1 has only one virtual frequency (−1481.11 cm^−1^), which is related to the stretching vibration of acetic acid-dissociated H^+^ between the C^1^ and O^2^ atoms. Finally, the product (VAc) was desorbed from B_12_N_12_NC, and the desorption energy was 15.9 kcal/mol. By comparing the reaction paths and energy changes in Figure 8, it is found that the system only needs to absorb 10.16 kcal/mol of energy from the outside to jump over the reaction energy barrier during the process of acetylene acetate reaction from the initial structure (Re+*) to the highest energy point (Ts1). Based on the method of determining the rate-control step proposed by Murdoch et al., the process from Co-ads1 to Ts1 is the rate-dependent step of acetylene acetate reaction catalyzed by B_12_N_12_NC in path 1, and the energy barrier of the rate-control step is 34.44 kcal/mol. 

In path 2, we found the transition state (Ts2) structure connecting Co-ads2 and Fs2 through a transition state search. During the formation of Ts2 from Co-ads2, the C^1^-C^2^ bond length of C_2_H_2_ changed from 1.340 Å to 1.417 Å, and the O-H bond on the carboxyl group of acetic acid broke (from 0.975 Å to 1.278 Å) to form free H^+^ between O^2^ and C^2^. The H^+^ dissociated from acetic acid attacks the C^2^ atom on B_12_N_12_NC-C_2_H_2_ to form B_12_N_12_NC-C_2_H_3_^+^ complexes, and the O^1^ atom on CH_3_COO^−^ attacks the C^1^ atom on B_12_N_12_NC-C_2_H_3_^+^ to form the final state (Fs) structure. Due to the difference of the C atoms attacked by H^+^, the product structure and reaction energy barrier formed by path 1 and path 2 are also different. The process system from Co-ads2 to Ts2 needs to absorb 37.48 kcal/mol of energy, and then the system releases 44.22 kcal/mol of energy from Ts2 to Fs2. The system needs to absorb 37.48 kcal/mol of energy in the process from Co-ads2 to Ts2, and then the system releases 44.22 kcal/mol of energy in the process from Ts2 to Fs2. Vibrational analysis shows that Ts1 has only one virtual frequency (−1472.17 cm^−1^), which is related to the stretching vibration of acetic acid-dissociated H^+^ between the C^2^ and O^2^ atoms. Finally, the product (VAc) was desorbed from B_12_N_12_NC, and the desorption energy was 5.41 kcal/mol. By comparing the reaction paths and energy changes in Figure 8, it is found that the system needs to absorb 20.38 kcal/mol of energy from the outside during the process of acetylene acetate acidification from the initial structure (Re+*) to the highest energy point (Ts2). Based on the method of determining the rate-control step proposed by Murdoch et al., the process from Co-ads2 to Ts2 is the rate-control step of the acetylene acetate reaction catalyzed by B_12_N_12_NC in path 2, and the energy barrier of the rate-control step is 37.48 kcal/mol.

From the adsorption energy of reactants, the adsorption energy of Co-ads1 is 7.18 kcal/mol lower than that of Co-ads2; from the total energy of the reaction process, the energy required for path 1 is 10.22 kcal/mol lower than that for path 2; from the single energy barrier of the speed control step, the energy barrier of path 1 is 3.04 kcal/mol lower than that of path 2. These data show that the structure in path 1 is more stable in thermodynamics under the reaction condition of 493.15 K, and the reaction path in path 1 is easier to proceed.

### 2.4. Source Analysis of Catalytic Activity Differences of Different Boron Nitride Nanomaterials

#### 2.4.1. Effects of Different Geometric Structures (Dimension) on Catalytic Properties of BN Nanomaterials

**Figure 9:**
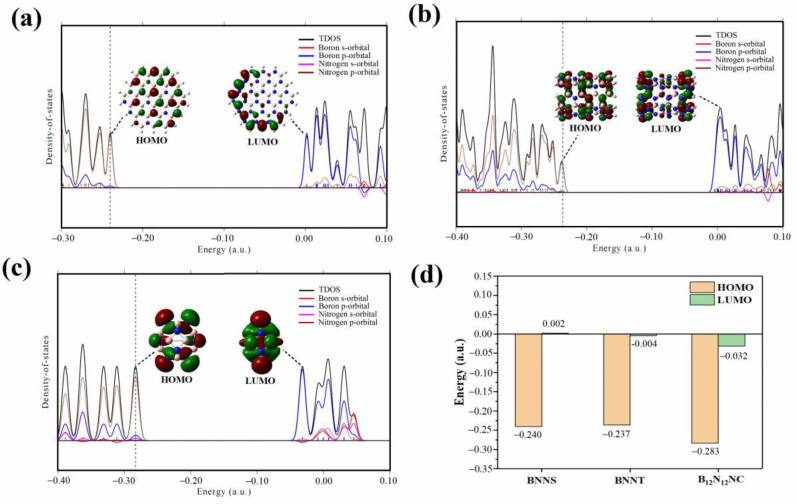
Density of state (DOS) plots of BNNS (**a**), BNNT (**b**), and BNNC (**c**) and their corresponding frontier molecular orbitals; (**d**) is the energy histogram of the HOMO orbitals and the LUMO orbitals of the three BN nanomaterials.

Firstly, we explored the reason why B_12_N_12_NC could form complexes with acetylene molecules, but BNNS and BNNT could not form complexes. As shown in (a–c) of Figure 9, we analyzed the density of states (DOS) of three optimized BN nanomaterials using the Multiwfn software package [37] and discussed the contribution of s and p orbitals of the B and N atoms in BN nanomaterials to the TDOS curve. The analysis shows that the highest occupied molecular orbital (HOMO) on the three BN nanomaterials is mainly contributed by the p orbital of N atoms, the lowest unoccupied molecular orbital (LUMO) is mainly contributed by the p orbital of B atoms. According to Koopmans’ theorem [38], the negative value of the HOMO energy level represents the first ionization energy of the substance. The lower the ionization energy is, the easier it is for the substance to lose electrons. The LUMO energy level is numerically equivalent to the electron affinity of the molecule. The lower the LUMO energy level, the easier it is to obtain electrons. Figure 9d shows the HOMO and LUMO values of BN nanomaterials with different structures. Among the three BN nanostructures, B_12_N_12_NC has the lowest HOMO energy level (the highest ionization energy) and the lowest LUMO energy level, which also indicates that B_12_N_12_NC is easier to obtain electrons. On the contrary, when acetylene molecules are adsorbed on the surface of B_12_N_12_NC, the easier it is for the C atom on acetylene to give electrons to form vinyl and further form stable complexes with B_12_N_12_NC.

**Figure 10:**
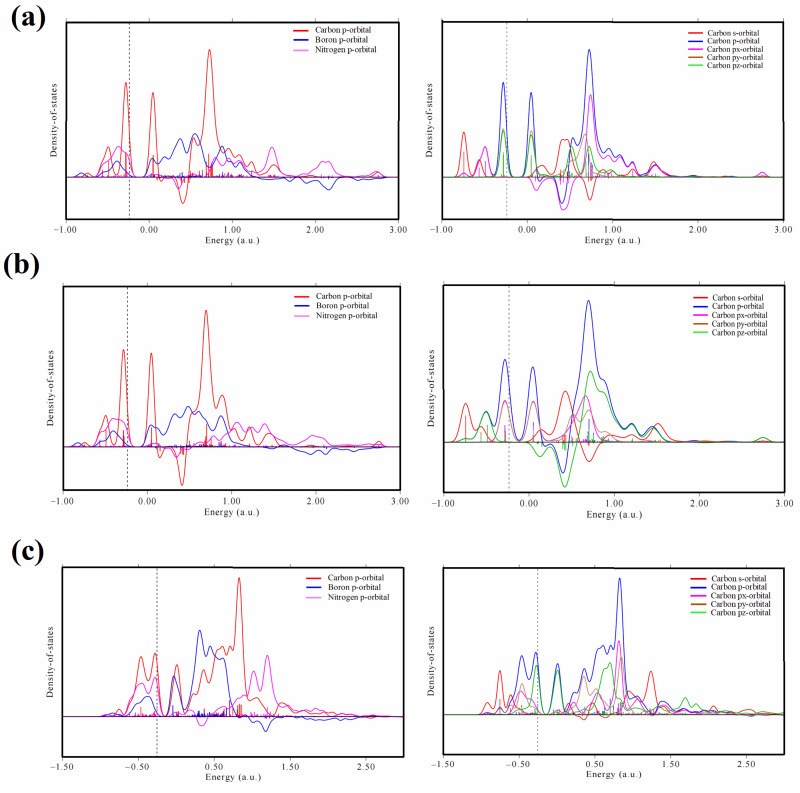
PDOS diagrams of acetylene adsorption structure on three different BN nanomaterials ((**a**) left, (**b**) left, (**c**) left are DOS diagrams of C, B, and N atom p orbitals in an acetylene single adsorption structure on BNNS, BNNT, and B_12_N_12_NC, respectively; (**a**) right, (**b**) right, (**c**) right are DOS diagrams of different C^1^ and C^2^ atom orbitals in an acetylene single adsorption structure on BNNS, BNNT, and B_12_N_12_NC, respectively).

By studying the density functional theory of BN nanomaterials with acetylene acetate reaction (dimension), we found that the key to acetylene acetate reaction catalyzed by BN nanomaterials is the formation of complexes between acetylene molecules and BN nanomaterials. As shown in (a) left, (b) left, and (c) left in Figure 10, orbital hybridization did not occur between the C atom on acetylene and the B, N atoms at adsorption sites in BNNS and BNNT. However, the p orbitals of the C atoms on B_12_N_12_NC overlap with those of the B and N atoms near the Fermi level, which indicates that C atoms form obvious orbital hybridization with B and N atoms, respectively, acetylene molecules change from the original C≡C bond to C=C bond, and the hybrid orbitals change from sp hybridization to sp^2^ hybridization. Because an sp^2^ hybrid has more hybrid orbitals, the overall energy of the outer electrons of the atom is lower at the same energy level, which makes the structure of B_12_N_12_NC-C_2_H_2_ complexes more stable in thermodynamics. From (a) right, (b) right, and (c) right in Figure 10, the p orbitals of C in sp hybrid acetylene are mainly contributed by two almost completely overlapping p orbitals near the Fermi level, while the p orbitals of C in sp^2^ hybrid B^12^N^12^BNs-C_2_H_2_ complexes are almost entirely contributed by p_z_ orbitals near the Fermi level, and the other two orbitals contribute very little to the p orbitals of the C atom. Since the two p orbitals of the π bond formed by acetylene overlap more than the p orbitals of the C atom in the B_12_N_12_NC-C_2_H_2_ complexes, the π bond formed is more stable, and the sp hybrid carbon atom in the acetylene molecule is closely combined with the outer valence electron (π electron), making it difficult to give electrons, thus making it difficult for the electrophilic attack of H^+^ dissociated by acetic acid. On the contrary, when acetylene molecules form complexes with BN nanomaterials, the hybridization mode of the C atoms on acetylene changes, the binding strength of C atoms to the outer valence electrons is weakened, and the electrophilic attack of acetic acid-dissociated H^+^ on the C atoms is easier. This process also shows that the activation process of the acetylene molecule is the key factor to improve the reaction between acetic acid molecules and acetylene in the process of acetylene acetate reaction, and changing the geometric structure of BN nanomaterials may be an effective way to improve the activation of acetylene.

#### 2.4.2. Effects of Different Reaction Sites on Catalytic Properties of BN Nanomaterials

**Figure 11:**
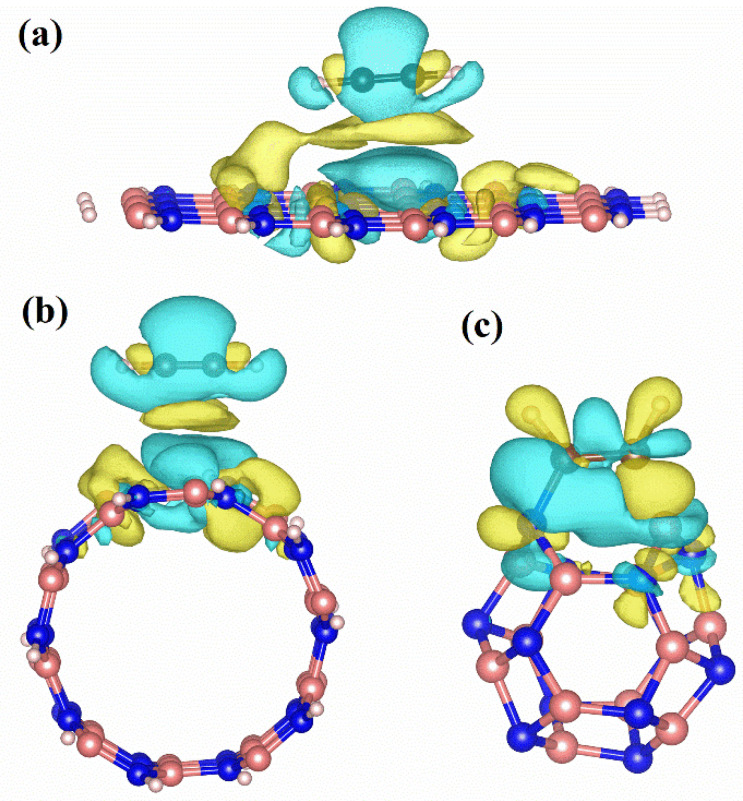
Electron density difference of BN nanomaterials with different geometric structures, where the yellow part is electron accumulation, and the blue part is electron depletion((**a**) the electron density of BN nanosheet (isosurface level: 0.001 e/Å^3^), (**b**) the electron density of BN nanotubes (isosurface level: 0.001 e/Å^3^), (**c**) the electron density of B_12_N_12_ nanocages (isosurface level: 0.01 e/Å^3^).

When acetylene forms complexes with BN nanomaterials, C atoms will bond with B and N atoms, respectively, which also leads to the difference between the two C atoms. Based on the reaction sites, we found that the reaction activities of acetic acid molecules attacking different C sites on B_12_N_12_NC-C_2_H_2_ complexes were also different. By comparing the two reaction paths on BNNCs, it can be seen that that H^+^ dissociated from acetic acid attacks C^1^ atom bonded with B atom, and O^1^ atom of carboxyl acetate attacks C^2^ atom bonded with N atom to produce vinyl acetate. In order to reveal the source of this difference, the adsorption structures of acetylene on three BN nanomaterials were analyzed using electron density difference (EDD). As shown in Figure 11, the yellow part is electron accumulation, and the blue part is electron depletion. It can be seen from the Figure 11 that the electron density transfer on B_12_N_12_NC is significantly greater than that on BNNS and BNNT, and the isosurface level of EDD on B_12_N_12_NC is ten times that on BNNS and BNNT. Therefore, we only conduct a detailed analysis based on the electron transfer on B_12_N_12_NC. The electron density transfer of the C atom on C_2_H_2_ is different from that of B and N atoms. The EDD diagram shows that electron depletion occurs near the C-N bond and electron aggregation occurs near the C-B bond. Therefore, the negatively charged O^1^ atom on CH_3_COO^−^ is more likely to attack the C^2^ atom on the C-N bond, and the positively charged H^+^ dissociated by acetic acid is more likely to attack the C^1^ atom on the C-B bond.

**Figure 12:**
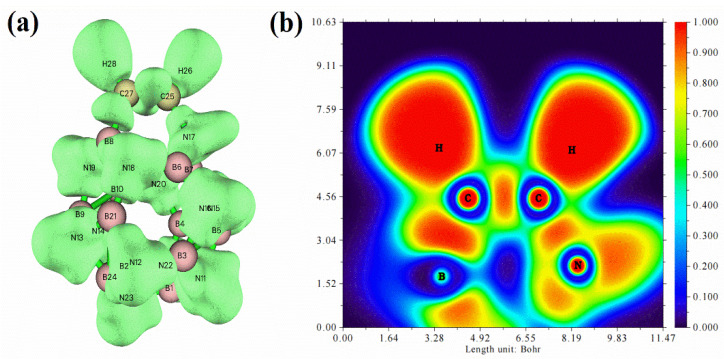
Electron localization function (ELF) of acetylene adsorbed on B_12_N_12_ nanocages ((**a**) is the global ELF diagram of acetylene adsorption structure on B_12_N_12_NC, (**b**) is the local ELF isosurface diagram of acetylene adsorption site).

In addition, we further determined that the reaction mode in path 1 is more favorable by the electron localization function (ELF) [39]. Figure 12a shows the global ELF diagram of the acetylene adsorption structure on B_12_N_12_NC, and Figure 12b shows the local ELF isosurface of acetylene adsorption sites. ELF can be considered as the position of spontaneous condensation of valence electrons due to the formation of lone pair electrons or covalent bonds. The smaller the ELF, the weaker the localization of electrons in this region. Comparing the ELF around two C atoms on acetylene in Figure 12, it can be seen that the valence electrons in the region around the C atom connected with N atom are relatively divergent. Since the carbon nucleus in this region is the least shielded by valence electrons, it is easier to be attacked by nucleophiles. This explains why there are differences in reaction energy barriers when acetic acid molecules attack different C sites.

## 3. Materials and Methods

All of the studies in this paper were conducted using the Becke, 3-parameter, Lee–Yang–Parr (B3LYP) functional of density functional theory (DFT) [40,41], and calculations were performed using the Gaussian 09 [42] software package. The B3LYP nonlocal correlation functional with the 6-31G(d,p) [43] basis set was applied to B, C, N, O, and H atoms. The DFT-D3 method [44] was used to calculate the density functional dispersion correction, and no symmetry constraints were assumed in the geometrical optimization. Considering the influence of reaction temperature and base group superposition error, the relative energy of all optimized configurations along the reaction path is the sum of electronic and thermal free energies (*G*) at the same optimization level. In addition, in order to be closer to the real reaction conditions, we used Shermo code [45] to calculate the Gibbs free energy (*G*) of each structure at 493.15 K. Gibbs free energy can be calculated as in Equation (1), where *G* is the Gibbs free energy of the system, *E_(elec)_* is the total electronic energy of the system, and *ZPE* is the zero-point energy of the system. *E_(vib)_*, *E_(rol)_*, and *E_(transl)_* are the vibrational energy, rotational energy, and translational energy of the system, respectively, and *S* is the entropy of the system. The calculation formulas of entropy (s) and zero-point energy (*ZPE*) are shown in Equations (2) and (3).

(1) $$G=E_{\left(elec\right)}+ZPE+E_{\left(vib\right)}+E_{\left(rol\right)}+E_{\left(trans\right)}+RT-TS$$

(2) $$S=Nk_{B}+Nk_{B}\ln\left(\frac{q\left(V,T\right)}{N}\right)+Nk_{B}T{\left(\frac{\partial \ln q}{\partial T}\right)}_{V}$$

(3) $$ZPE=\frac{\sum hv}{2}$$

In this work, all structural (reactant, product, intermediate, or transition state structure in the reaction process) calculations are carried out at the same calculation level. Through the harmonic vibration frequency, we verify that the optimized geometry has no frequency, and all eigenvalues of its force constant matrix are positive, so as to ensure that the structure is the minimum stable point on the energy plane. Similarly, by calculating the vibration frequency of the transition state structure, it is found that there is a unique virtual frequency and the eigenvalue of the force constant matrix is only negative. However, the correct transition state (Ts) does not only have a virtual frequency, and the vibration direction of this virtual frequency is related to the desired reactants and products. Therefore, we calculate the intrinsic reaction coordinates (IRC) [46,47] of the transition state structure, and verify the reactants and products connected at both ends of Ts, so as to determine the correct transition state structure. During this study, the method of calculating adsorption energy and co-adsorption energy is shown in Equations (4) and (5), where *G_ads-state_* and *G_co-ads-state_* represent the total Gibbs free energy of the adsorbed substance and catalyst, and *G_Catalyst_*, *G*_*C*_2___*H*_2__, and *G*_*CH*_3__*_COOH_* are the Gibbs free energy of a single catalyst and a single reactant, respectively. The process of calculating the energy barrier of the reaction rate-control step on each reaction path adopts the method of finding the rate-control step proposed by Murdoch et al. [35].

(4) $$\Delta G_{ads}=G_{ads-state}-\left(G_{C_{2}H_{2}/}{}_{CH_{3}COOH}+G_{Catalyst}\right)$$

(5) $$\Delta G_{Co-ads}=G_{Co-ads-state}-\left(G_{C_{2}H_{2}}+G_{CH_{3}COOH}+G_{Catalyst}\right)$$

## 4. Conclusions

In this work, we studied the catalytic reaction of acetylene and acetic acid with boron nitride nanomaterials with an acetylene acetate reaction. The energy barriers of the rate-control steps of the acetylene acetate reaction catalyzed by BN nanomaterials are arranged from small to large as follows: BNNC < BNNT < BNNS. Based on the geometric configuration (dimension), we found that the catalytic performance of the cage structure is significantly better than that of the tubular and sheet structure in BN materials. The source of its special activity was the chemisorption of acetylene by B_12_N_12_ nanocages. Compared with the physical adsorption of BNNT and BNNS on the reactants, the B_12_N_12_NC-C_2_H_2_ complexes are formed between acetylene molecules and the B_12_N_12_NC-like catalyst through chemical adsorption. In the process of the acetylene forming complexes, the C≡C bond (sp hybrid) is transformed into a C=C bond (sp^2^ hybrid), which activates and polarizes the acetylene molecule, thus improving the reaction activity with the acetic acid molecule. Based on the reaction sites, we found that the reaction activities of acetic acid molecules attacking different C sites on the B_12_N_12_NC-C_2_H_2_ complexes were also different. Through the analysis of EDD and ELF, it was proved that the C atom connected with B was more likely to have an electrophilic reaction with H^+^, and the C atom connected with N was more likely to have a nucleophilic reaction with CH_3_COO^−^, which further determined the more favorable attack mode of acetic acid molecules. Compared with 2D BNNS and 1D BNNT, 0D B_12_N_12_NC breaks the inherent reaction inertia of BN materials and shows excellent catalytic activity for the acetylene acetate reaction. It is very likely to be a potential non-metallic catalyst for the acetylene gas-phase preparation of vinyl acetate.

## Data Availability

The data presented in this study are contained within the article or are available upon request from the corresponding author Lihua Kang.

## Supplementary Materials

The following supporting information can be downloaded at: https://www.mdpi.com/article/10.3390/ijms23179997/s1.
