# Supplementary material for: Density Functional Theory Study of Low-Dimensional (2D, 1D, 0D) Boron Nitride Nanomaterials Catalyzing Acetylene Acetate Reaction
Source: Int J Mol Sci. 2022 Sep 2;23(17):9997. doi: 10.3390/ijms23179997 (PMC9456482; doi:10.3390/ijms23179997)
Supplement: Supplementary file 1 [file ijms-23-09997-s001.zip › ijms-1846008-supplementary.pdf]

Supporting Information

Density Functional Theory Study of Low-Dimensional (2D, 1D, 0D) Boron Nitride Nanomaterials Catalyzing Acetylene Acetate Reaction

Xunchao Zhang [1,2], Lihua Kang [1,*] and Mingyuan Zhu [2]

[1] College of Chemistry & Chemical Engineering, Yantai University, Yantai 264005, China; 18438591035@163.com
[2] School of Chemistry & Chemical Engineering, Shihezi University, Shihezi 832000, China; zhuminyuan@shzu.edu.cn
* Correspondence: lhkang@ytu.edu.cn

Catalyst and adsorption site	ΔG_{C2H2} (kcal/mol)	$\Delta G_{CH3COOH}$ (kcal/mol)	$\Delta G_{Co\text{-}adsorption}$ (kcal/mol)
BNNS-surface	2.68	4.57	8.05
BNNT-surface	4.39	7.65	12.87
BNNC-surface	-26.33	-19.43	-24.28
BNNS-boundary	3.01	4.38	9.71
BNNT-boundary	6.51	6.32	12.87

Table S1. Under the reaction conditions of 1atm and 493.15 K, the adsorption energies of acetylene and acetic acid on the surface and boundary positions of catalysts with different BN structures.

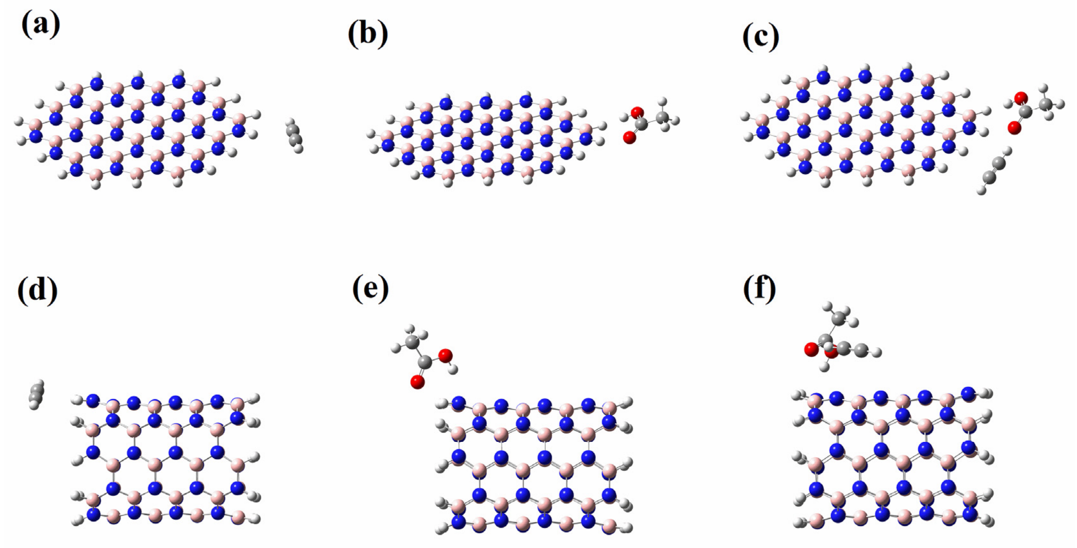

Figure S1. The optimized adsorption configurations of acetylene and acetic acid on BN nanosheets and BN nanotubes at the boundary positions closed by hydrogen atoms ((a), (b), (c) respectively represent the configurations of acetylene, acetic acid and CO adsorption on BNNS; (d) , (e), (f) represent the configurations of acetylene, acetic acid and CO adsorption on BNNT, respectively).
